# Supplementary material for: Proof-of-Concept for the Analgesic Effect and Thermoregulatory Safety of Orally Administered Multi-Target Compound SZV 1287 in Mice: A Novel Drug Candidate for Neuropathic Pain
Source: Biomedicines. 2021 Jun 29;9(7):749. doi: 10.3390/biomedicines9070749 (PMC8301340; doi:10.3390/biomedicines9070749)
Supplement: Supplementary file 1 [file biomedicines-09-00749-s001.zip › biomedicines-1217914-SI.pdf]

biomedicines

MDPI

Supplementary Materials

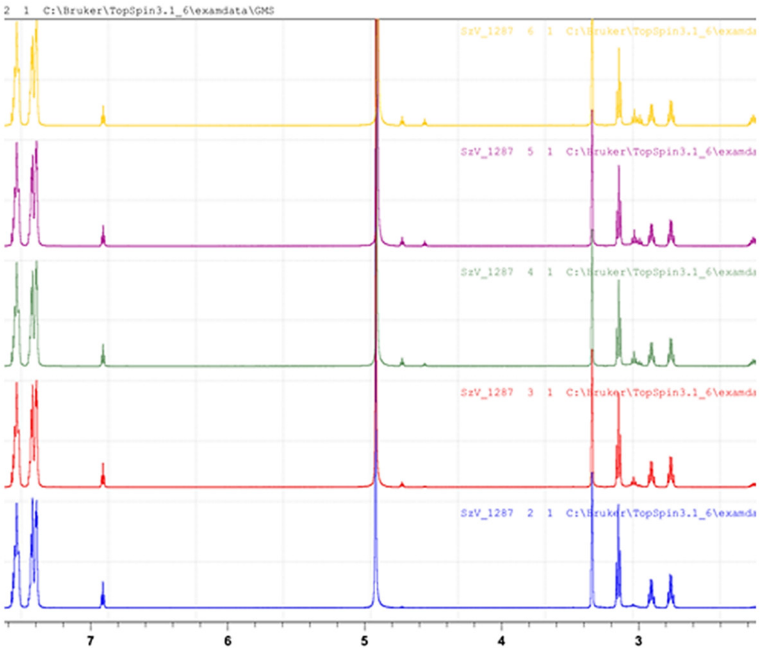

Figure S1. Series of ^{1}H NMR spectra of SZV 1287 in methanol–d4 (CD3OD) and deuterium oxide (D2O) + trifluoroacetic acid (TFA) taken between 5–120 min after treatment with trifluoroacetic acid (TFA).

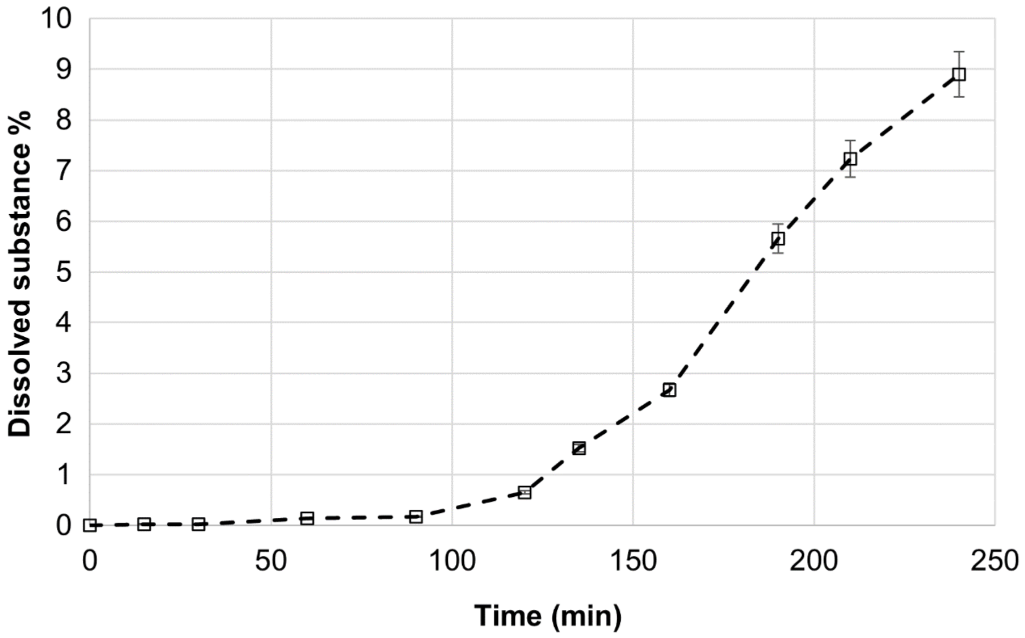

Figure S2. In vitro dissolution graph for SZV 1287 in pH 1.2 hydrochloric acid (0–120 min) and pH 6.8 phosphate buffer (120–240 min).

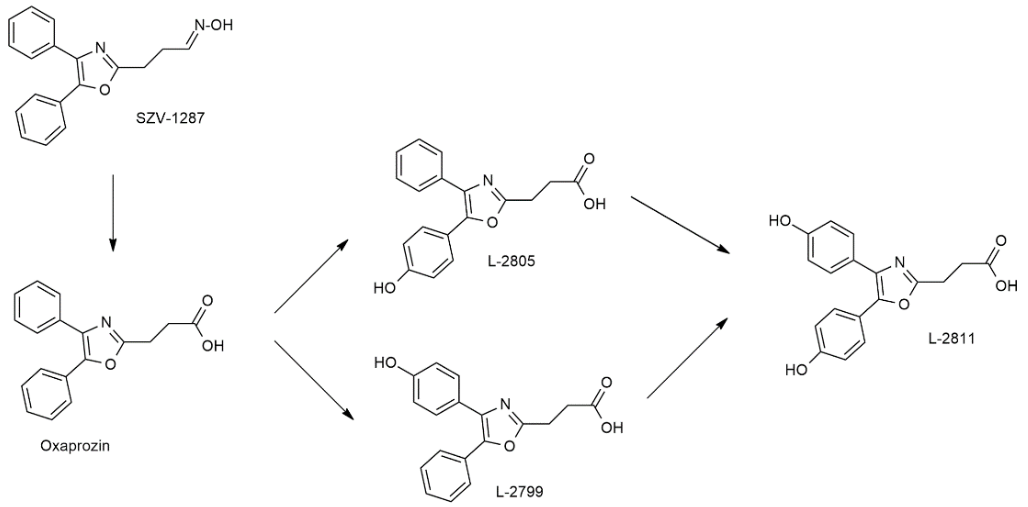

Figure S3. A potential route of formation of SZV 1287 metabolites. SZV 1287, 3-(4,5-diphenyl-1,3-oxazol-2-yl)propanal oxime; oxaprozin, 3-(4,5-diphenyloxazol-2-yl)propanoic acid; L 2799, 3-(4-(4-hydroxyphenyl)-5-phenyloxazol-2-yl)propanoic acid; L 2805, 3-(5-(4-hydroxyphenyl)-4-phenyloxazol-2-yl)propanoic acid; L 2811, 3-(4,5-bis(4-hydroxyphenyl)oxazol-2-yl)propanoic acid.

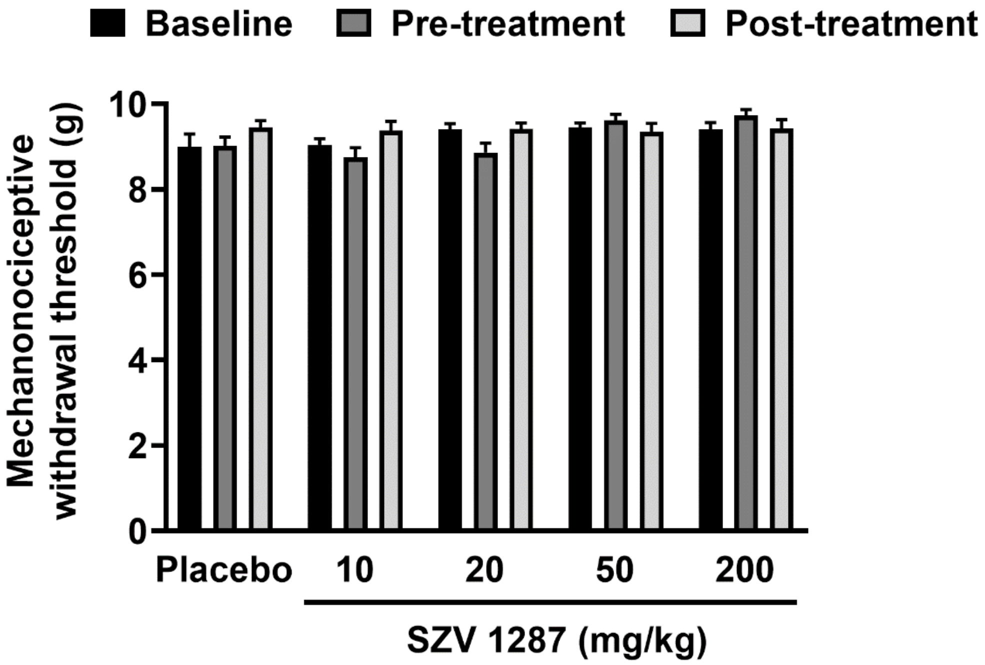

Figure S4. Mechanonociceptive withdrawal thresholds of the contralateral intact hind paws 2 h following single enterosolvent SZV 1287 capsule treatment (10, 20, 50 and 200 mg/kg) on 7th postoperative day. Data are shown as means ± S.E.M. of n = 7–14 mice/group (repeated measures two-way ANOVA followed by Tukey’s multiple comparison test).

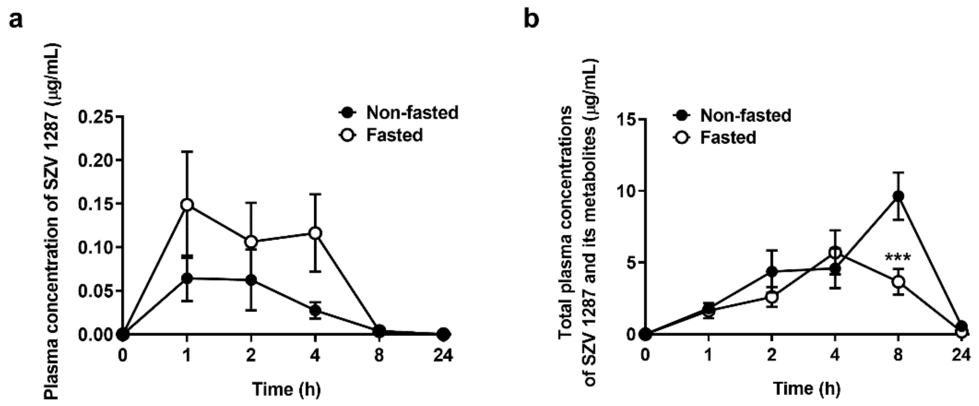

Figure. S5. Plasma concentrations-time curve of (**a**) SZV 1287, as well as (**b**) SZV 1287 and its main metabolites (oxaprozin, L 2799, L 2805, and L 2811) following enterosolvent capsule administration (20 mg/kg) in mice. Data are shown as means ± S.E.M. of n = 7-8 mice/time point, *** $p < 0.001$ vs. non-fasted group; ordinary two-way ANOVA followed (fasting factor F = 4.78, $p = 0.0316$) by Sidak's multiple comparison test.

Table Legends:

Table S1. Plasma concentrations of SZV 1287 and its main metabolites (L 2811, L 2805, L 2799, and oxaprozin) following single enterosolvent capsule treatment (10 mg/kg) in mice.

Animal Number	L 2811 (µg/mL)	L 2805 (µg/mL)	L 2799 (µg/mL)	Oxaprozin (µg/mL)	SZV 1287 (µg/mL)
1	ND	0.73	ND	1.56	ND
2	ND	ND	ND	ND	ND
3	ND	ND	ND	<0.5	ND
4	1.20	ND	ND	ND	ND
5	ND	<0.5	ND	1.21	ND

Abbreviation: ND, not detected with HPLC-UV.

Table S2. Plasma concentrations of SZV 1287 and its main metabolites (L 2811, L 2805, L 2799, and oxaprozin) following single enterosolvent capsule treatment (20 mg/kg) in mice.

Animal Number	L 2811 (µg/mL)	L 2805 (µg/mL)	L 2799 (µg/mL)	Oxaprozin (µg/mL)	SZV 1287 (µg/mL)
6	ND	ND	ND	1.78	ND
7	0.77	ND	ND	1.30	ND
8	ND	1.27	0.62	4.29	ND
9	ND	1.42	0.90	13.86	ND
10	ND	ND	ND	4.32	ND
11	ND	0.63	ND	1.14	ND
12	ND	0.71	ND	2.25	ND
13	ND	0.74	ND	3.87	ND
14	1.67	ND	ND	ND	ND

Abbreviation: ND, not detected with HPLC-UV.

Table S3. Plasma concentrations of SZV 1287 and its main metabolites (L 2811, L 2805, L 2799, and oxaprozin) following single enterosolvent capsule treatment (50 mg/kg) in mice.

Animal Number	L 2811 (µg/mL)	L 2805 (µg/mL)	L 2799 (µg/mL)	Oxaprozin (µg/mL)	SZV 1287 (µg/mL)
15	ND	0.59	ND	1.95	ND
16	ND	ND	ND	ND	ND
17	ND	1.44	0.73	10.40	ND
18	0.83	ND	ND	1.30	0.34
19	0.70	2.14	1.00	9.03	0.56
20	ND	ND	ND	1.43	ND
21	ND	0.72	ND	5.48	ND
22	ND	ND	ND	ND	ND

Abbreviation: ND, not detected with HPLC-UV.

Table S4. Plasma concentrations of SZV 1287 and its main metabolites (L 2811, L 2805, L 2799, and oxaprozin) following single enterosolvent capsule treatment (200 mg/kg) in mice.

Animal Number	L 2811 (µg/mL)	L 2805 (µg/mL)	L 2799 (µg/mL)	Oxaprozin (µg/mL)	SZV 1287 (µg/mL)
23	ND	<0.5	<0.5	1.4	<0.5
24	ND	2.6	0.7	9.7	<0.5
25	ND	2.6	0.7	10.9	<0.5
26	ND	2.8	0.7	6.6	<0.5
27	ND	3.5	1.0	14.0	0.9
28	ND	2.5	0.6	8.6	<0.5
29	2.0	1.9	0.6	10.9	0.7

Abbreviation: ND, not detected with HPLC-UV.

Table S5. Concentrations of SZV 1287 and its main metabolites (L 2811, L 2805, L 2799, and oxaprozin) in the brain following single enterosolvent capsule treatment (10 mg/kg) in mice.

Animal Number	L 2811 (µg/g)	L 2805 (µg/g)	L 2799 (µg/g)	Oxaprozin (µg/g)	SZV 1287 (µg/g)
1	ND	ND	ND	ND	ND
2	ND	ND	ND	ND	ND
3	ND	ND	ND	ND	ND
4	ND	ND	ND	ND	ND
5	ND	ND	ND	ND	ND

Abbreviation: ND, not detected with HPLC-UV.

Table S6. Concentrations of SZV 1287 and its main metabolites (L 2811, L 2805, L 2799, and oxaprozin) in the brain following single enterosolvent capsule treatment (20 mg/kg) in mice.

Animal Number	L 2811 (µg/g)	L 2805 (µg/g)	L 2799 (µg/g)	Oxaprozin (µg/g)	SZV 1287 (µg/g)
6	<0.4	ND	ND	ND	<0.4
7	ND	ND	ND	ND	ND
8	<0.4	ND	ND	<0.4	<0.4
9	<0.4	ND	ND	<0.4	0.41
10	<0.4	ND	ND	<0.4	ND
11	ND	ND	ND	ND	0.66
12	ND	ND	ND	ND	<0.4
13	ND	ND	ND	ND	ND
14	ND	ND	ND	ND	ND

Abbreviation: ND, not detected with HPLC-UV.

Table S7. Concentrations of SZV 1287 and its main metabolites (L 2811, L 2805, L 2799, and oxaprozin) in the brain following single enterosolvent capsule treatment (50 mg/kg) in mice.

Animal Number	L 2811 (µg/g)	L 2805 (µg/g)	L 2799 (µg/g)	Oxaprozin (µg/g)	SZV 1287 (µg/g)
15	<0.4	ND	ND	ND	ND
16	<0.4	ND	ND	ND	ND
17	<0.4	ND	ND	<0.4	<0.4
18	<0.4	ND	ND	ND	0.94
19	ND	ND	ND	<0.4	1.16
20	ND	ND	ND	ND	ND
21	ND	ND	ND	<0.4	<0.4
22	ND	ND	ND	ND	ND

Abbreviation: ND, not detected with HPLC-UV.

Table S8. Concentrations of SZV 1287 and its main metabolites (L 2811, L 2805, L 2799, and oxaprozin) in the brain following single enterosolvent capsule treatment (200 mg/kg) in mice.

Animal Number	L 2811 (µg/g)	L 2805 (µg/g)	L 2799 (µg/g)	Oxaprozin (µg/g)	SZV 1287 (µg/g)
23	ND	ND	ND	ND	ND
24	ND	ND	ND	ND	1.1
25	ND	ND	ND	<0.4	1.1
26	ND	ND	ND	<0.4	0.9
27	ND	ND	ND	0.5	4.1
28	ND	ND	ND	<0.4	0.7
29	ND	ND	ND	0.4	1.7

Abbreviation: ND, not detected with HPLC-UV.
